# Arabidopsis translation initiation factor binding protein CBE1 negatively regulates accumulation of the NADPH oxidase respiratory burst oxidase homolog D

**DOI:** 10.1016/j.jbc.2023.105018

**Published:** 2023-07-07

**Authors:** Jeoffrey George, Martin Stegmann, Jacqueline Monaghan, Julia Bailey-Serres, Cyril Zipfel

**Affiliations:** 1 The Sainsbury Laboratory, University of East Anglia, Norwich Research Park, Norwich, United Kingdom; 2 Institute of Plant and Microbial Biology and Zürich-Basel Plant Science Center, University of Zürich, Zürich, Switzerland; 3 Department of Botany and Plant Sciences, Center for Plant Cell Biology, University of California, Riverside, Riverside, California, USA

**Keywords:** innate immunity, signaling, ROS, translation, NADPH oxidase

## Abstract

Cell surface pattern recognition receptors sense invading pathogens by binding microbial or endogenous elicitors to activate plant immunity. These responses are under tight control to avoid excessive or untimely activation of cellular responses, which may otherwise be detrimental to host cells. How this fine-tuning is accomplished is an area of active study. We previously described a suppressor screen that identified *Arabidopsis thaliana* mutants with regained immune signaling in the immunodeficient genetic background *bak1-5*, which we named *modifier of bak1-5* (*mob*) mutants. Here, we report that *bak1-5 mob7* mutant restores elicitor-induced signaling. Using a combination of map-based cloning and whole-genome resequencing, we identified MOB7 as conserved binding of eIF4E1 (CBE1), a plant-specific protein that interacts with the highly conserved eukaryotic translation initiation factor eIF4E1. Our data demonstrate that CBE1 regulates the accumulation of respiratory burst oxidase homolog D, the NADPH oxidase responsible for elicitor-induced apoplastic reactive oxygen species production. Furthermore, several mRNA decapping and translation initiation factors colocalize with CBE1 and similarly regulate immune signaling. This study thus identifies a novel regulator of immune signaling and provides new insights into reactive oxygen species regulation, potentially through translational control, during plant stress responses.

The restriction of invading organisms is governed by passive and active defenses, which are effective against all types of plant pathogens and pests, including viruses, insects, nematodes, and parasitic plants (1). On the cell surface, conserved microbial molecules called pathogen- or microbe-associated molecular patterns or plant-derived damage-associated molecular patterns and phytocytokines (hereafter, generally referred to as elicitors) are recognized by pattern recognition receptors (PRRs) (2, 3). For example, in *Arabidopsis thaliana* (hereafter Arabidopsis), the PRRs flagellin sensing 2 (FLS2), EF-TU receptor, and PEP1 receptor 1 and PEP1 receptor 2 recognize bacterial flagellin (and its cognate ligand, flg22), bacterial EF-Tu (and its cognate ligand, elf18), and endogenous Atpep1 and related peptides, respectively (4, 5, 6). These PRRs interact with the common coreceptor brassinosteroid insensitive 1-associated kinase 1 (BAK1) in a ligand-dependent manner (7, 8, 9). Following heterodimerization, numerous cell signaling events are initiated, including activation of receptor-like cytoplasmic kinases, production of apoplastic reactive oxygen species (ROS) catalyzed by the NADPH oxidase respiratory burst oxidase homolog D (RBOHD), altered ion fluxes, activation of calcium-dependent protein kinases, mitogen-activated protein kinase (MAPK) cascades, callose deposition, and large-scale transcriptional programming (10, 11). To maintain immune homeostasis, plants use multiple strategies to adjust the amplitude and duration of immune responses (11). These include limiting the ability of PRRs to recruit their cognate coreceptors, regulation of signaling initiation and amplitude at the level of PRR complexes (*i.e.*, post-translational modifications, protein turnover), monitoring of cytoplasmic signal-transducing pathways, and control of transcriptional reprogramming (11).

To identify loci involved in plant immunity, we previously conducted a forward genetic screen in the immunodeficient mutant *bak1-5*, called the *modifier of bak1-5* (*mob*) screen (12). This ethyl methanesulfonate (EMS)-induced suppressor screen of *bak1-5* phenotypes identified 10 mutants in nine allelic groups, named *mob1* to *mob10*, with partially restored elicitor-induced ROS production (12, 13, 14). Through this suppressor screen, novel regulators of immune signaling have been discovered. *MOB1* and *MOB2* encode calcium-dependent protein kinase 28, which negatively regulates immune signaling by controlling the accumulation of the receptor-like cytoplasmic kinase botrytis-induced kinase 1, a central kinase involved in immune signaling downstream of multiple PRRs (12, 15, 16). *MOB4* encodes constitutive active defense 1 (13). Constitutive active defense 1 is involved in immunity at different levels by controlling programmed cell death and regulating the homeostasis of the phyllosphere microbial community (17, 18). MOB6 corresponds to site-1 protease, which controls the maturation of the endogenous rapid alkalinization factor 23 peptide to regulate immune signaling *via* the receptor kinase FERONIA (14, 19, 20). Hence, we predict that the identification of remaining *MOB* genes will continue to unravel mechanisms of immune regulation.

Here, we report that MOB7 corresponds to conserved binding of eIF4E1 (CBE1), a plant-specific protein that associates with the 5′ mRNA cap (21) and the translation initiation factor eIF4E1 (22). We show that CBE1 colocalizes with ribonucleoprotein complexes and that *cbe1* and other translational regulator mutants display enhanced accumulation of RBOHD protein, resulting in enhanced antibacterial immunity and ROS production, possibly through translational control of RBOHD protein levels.

## Results

### The *mob7* mutation rescues *bak1-5* immunodeficiency

**Figure 1:**
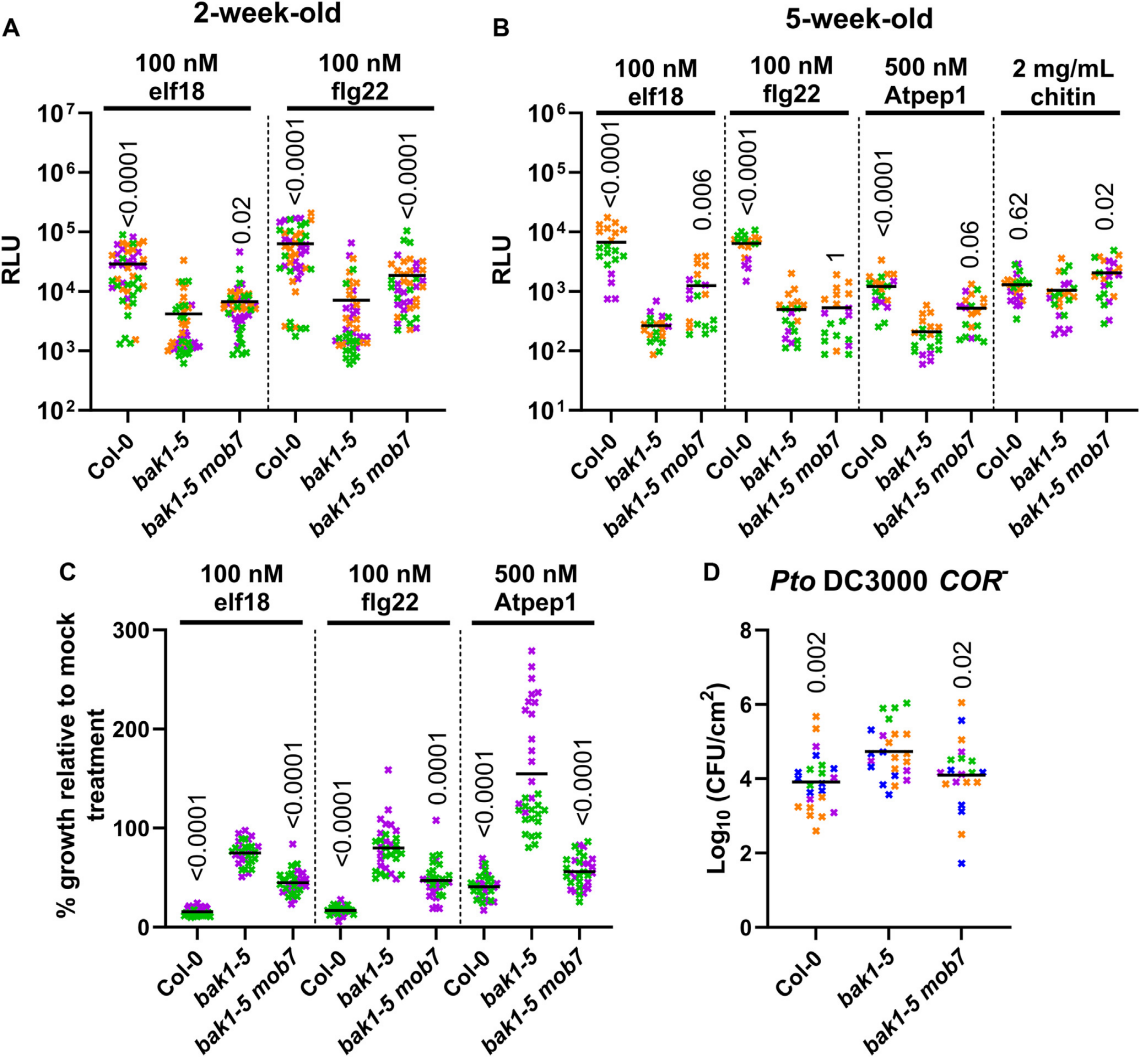
***mob7* restores immune signaling in *bak1-5*.***A* and *B*, total ROS accumulation measured as relative light units (RLU) over 60 min recording after treatment with the corresponding elicitors on (*A*) 2-week-old seedlings (n = 12–16) or (*B*) leaf discs from leaves of 5-week-old plants (n = 4–8). *Horizontal lines* represent the means from three independent experiments (n = 4–8). *C*, growth inhibition is represented as relative fresh weight compared to untreated seedlings in response to the indicated elicitors. *Horizontal lines* represent the means from two independent experiments (n = 12–17). *D*, bacterial growth (colony-forming units—cfu/cm^2^) in leaves spray-inoculated with 10^7^ cfu/ml (*A*_600_ = 0.2) *P. syringae* pv. *tomato* (Pto) DC3000 *COR*^*-*^ and sampled at 3 dpi. *Horizontal lines* represent the means from four independent experiments (n = 4–8). (*A*–*D*) *Symbol* colors indicate different experiments. Numbers above symbols are *p*-values from (*A*, *B* and *C*) Dunn’s or (*D*) Dunnett’s multiple comparison test between corresponding genotypes and *bak1*-*5*. ROS, reactive oxygen species.

In the present study, we describe and characterize the *mob7* mutation. First, we confirmed that the *mob7* mutation was maintained in the M_5_ generation, as *bak1-5 mob7* suppressor mutants displayed partially restored ROS (H_2_O_2_) production in seedlings upon treatment with the elicitors elf18 and flg22 (Fig. 1*A*). In addition, the *mob7* mutation increased ROS production in adult leaves upon elicitation with elf18, Atpep1, and chitin; however, no difference was observed with flg22 (Figs. 1*B* and S1, *A*–*D*). Despite partially rescuing the ROS phenotype quantitatively, the *mob7* mutation did not restore the delayed peak of ROS burst observed in *bak1-5* (Fig. S1, *B* and *E*). However, the delayed response observed in *bak1-5* is thought to be due to the compensation by other SERKs (23), which might not be as active as SERK3/BAK1 in immune signaling. This phenotype suggests a role of CBE1 downstream of the SERKs.

A late immune output triggered by several elicitors is the inhibition of seedling growth (10). While seedling growth inhibition is largely blocked in the *bak1-5* mutant (9, 23), it was restored in suppressor mutant *bak1-5 mob7* upon prolonged exposure with elf18, flg22, or Atpep1, while mock-treated seedlings grew similar to wildtype (WT) Col-0 (Figs. 1*C* and S1*F*). This sensitivity to flg22 of the *bak1-5 mob7* mutant during seedling development, which was not observed in adult leaves to induce a partial regain of ROS production compared to *bak1-5*, is likely due to the different expression level of *FLS2* at various developmental stage (24, 25), and different growth conditions as some hormones regulate *FLS2* expression and consequently flg22-triggered responses (26, 27, 28, 29). Furthermore, immunity to the hypovirulent bacterial strain *Pseudomonas syringae* pathovar *tomato* (*Pto*) DC3000 *COR*^*-*^ was restored in *bak1-5 mob7* suppressor mutants compared to *bak1-5* (Fig. 1*D*). Altogether, these results show that *mob7* partially restores immunity in *bak1-5*.

### Identification of *MOB7* as *CBE1*

**Figure 2:**
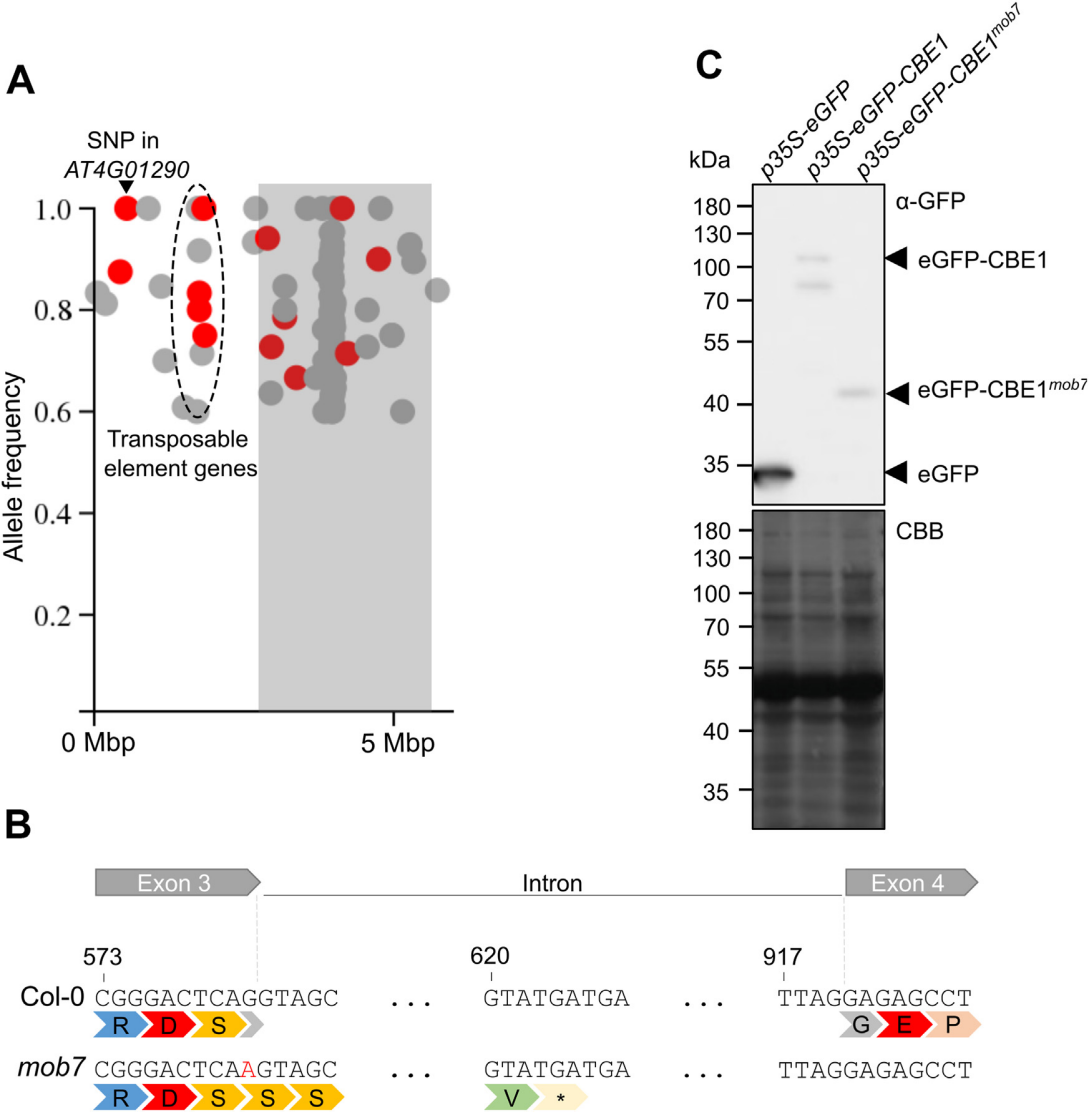
***mob7* mutation maps to *conserved binding of eIF4E1* resulting in a truncated protein.***A*, density plot of SNPs at the *top* arm of chromosome 4 using CandiSNP software (Etherington *et al.*, 2014). SNPs with an allele frequency below 60% were removed from the plots. Nonsynonymous SNPs are shown in *red* and others in *gray*. *Gray rectangles* indicate the centromere. The *dashed area* delimits several nonsynonymous SNPs in transposable element genes. *B*, the *mob7* mutation leads to a premature stop codon within the intron downstream of exon 3. The *top* symbols delimit nucleotides from exons 3, 4 and intron within *AT4G01290*. The number indicates the nucleotide position relative to the adenosine of the start codon. The second line shows amino acids corresponding to codons above. The EMS-induced SNP in *mob7* is indicated in *red*. *Star* indicates a stop codon. *C*, immunoblot analysis using anti-GFP after transient expression in *N. benthamiana*. Coomassie Brilliant *Blue* stain is shown as loading control. Experiment was repeated once with similar results. Mbp, mega base pairs; SNP, single-nucleotide polymorphism.

Using the elicitor-induced ROS phenotype of *mob7* and map-based cloning of the F_2_ population from the outcross of *bak1-5 mob7* (Col-0 ecotype) with L*er*-0, linkage analysis revealed three regions of interest (Fig. S2). Whole-genome resequencing of bulked F_2:3_ segregants that rescued seedling growth inhibition upon 1 μM Atpep1 treatment identified a single nucleotide polymorphism in *AT4G01290*, a gene that encodes CBE1 (Fig. 2*A*). The G to A transition is located at the last nucleotide of the third exon (Fig. 2*B*), which leads to a premature stop codon. This results in reduced *CBE1* expression (Fig. S3, *B* and *C*). In addition, transient expression of eGFP-CBE1^*mob7*^ in *Nicotiana benthamiana* revealed a truncated protein with an apparent molecular weight of 44 kDa, while GFP-CBE1 migrated at 137 kDa (Figs. 2*C* and S3*A*). The discrepancy of size observed and additional bands may be caused by yet unknown posttranslational modifications of CBE1 (Fig. 2*C*). It is possible that the premature stop codon in *mob7* is recognized by the nonsense-mediated mRNA decay (NMD) machinery, which links premature translation termination to mRNA degradation (30).

**Figure 3:**
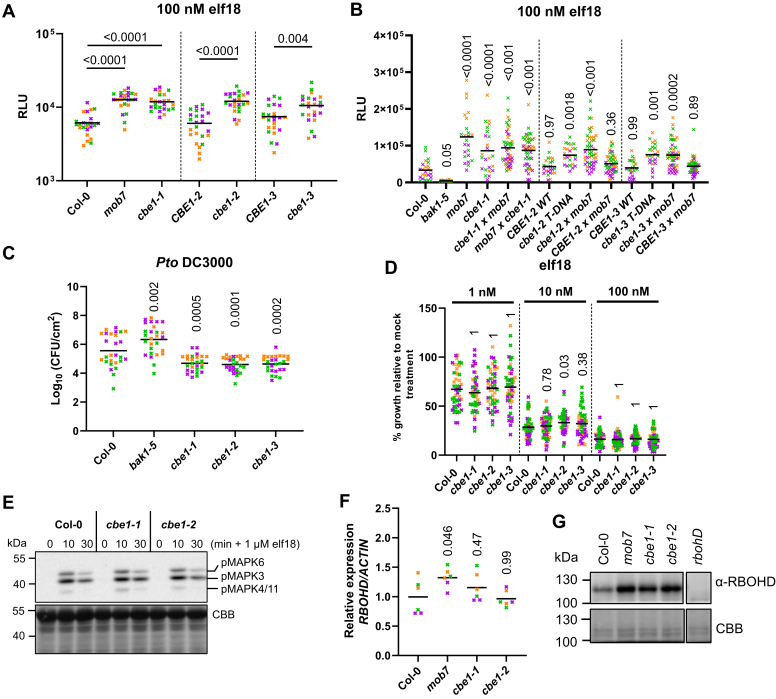
**CBE1 negatively regulates elicitor-induced ROS production andRBOHD protein levels.***A* and *B*, total ROS accumulation measured as RLU over 60 min recording after treating leaf discs from 5-week-old plants with 100 nM elf18. *Horizontal lines* represent the means from three independent experiments (n = 8). *C*, bacterial growth (CFU/cm^2^) in leaves spray inoculated with 10^7^ CFU/ml (*A*_600_ = 0.2) *P. syringae* pv. *tomato* DC3000 and sampled at 3 dpi. *Horizontal lines* represent the means from three independent experiments (n = 9). *D*, growth inhibition represented as percentage of fresh weight in response to 1, 10, or 100 nM elf18 relative to mock treated seedlings. *Horizontal lines* represent the means from three independent experiments (n = 16). *E*, immunoblot analysis of elf18-induced MAPK phosphorylation using anti-phospho-p44/42 in leaf discs from leaves of 5-week-old plants treated with 1 μM elf18 for the indicated time. Coomassie Brilliant Blue (CBB) stain is shown as loading control. Experiment was repeated twice with similar results. *F*, qRT-PCR of *RBOHD* transcripts in leaf discs from 5-week-old plants. Expression values are relative to *ACTIN2*. *Horizontal lines* represent the means from three independent experiments (n = 2). *G*, immunoblot analysis of RBOHD (anti-RBOHD) and BAK1 (anti-BAK1) protein accumulations in 5-week-old Arabidopsis leaves from corresponding genotypes. CBB stain is shown as loading control. Experiment was repeated twice with similar results. Symbol *colors* indicate different experiments. Numbers above symbols are *p*-values from (*A*, *B*, *C* and *F*) Dunnett’s or (*D*) Dunn’s multiple comparison test between corresponding genotypes or (*B*, *C*, *D*, and *F*) Col-0. BAK1, brassinosteroid insensitive 1-associated kinase 1; CBE1, conserved binding of eIF4E1; RBOHD, respiratory burst oxidase homolog D; RLU, relative light units; ROS, reactive oxygen species.

Knock-down alleles from independent T-DNA insertions with reduced *CBE1* expression phenocopied the increased elf18-induced ROS production and normal growth observed in *mob7* single mutant (Figs. 3*A* and S3, *A*–*D*), while WT segregants from the T-DNA alleles *cbe1-2* and *cbe1-3*, named *CBE1-2* and *CBE1-3* respectively, have the same phenotype as Col-0 (Figs. 3*A* and S3, *A*–*C*). Moreover, F_1_ progeny from *mob7* crossed with two independent T-DNA mutant alleles, *cbe1-2* or *cbe1*-*3*, retained hypersensitivity to elf18, while F_1_ progeny from *mob7* crossed to *CBE1-2* or *CBE1-3* wildtype segregants did not (Fig. 3*B*), indicating that *mob7* and *cbe1* are allelic. This confirms that the *mob7* phenotype is caused by a mutation in *CBE1*.

### CBE1 is a negative regulator of elicitor-induced ROS production and immunity

While mutation of *CBE1* results in increased ROS production induced by various elicitors (Figs. 3*A* and S4*A*) and enhanced immunity to *Pto* DC3000 *COR*^*-*^ (Fig. 3*C*), we did not observe any difference in seedling growth inhibition or MAPK activation between different *cbe1* alleles and Col-0 (Figs. 3, *D* and *E* and S4*B*). Given the apparent specific impact of *cbe1* mutations on ROS production, we tested whether transcripts and/or protein levels for the NADPH oxidase RBOHD were affected. Interestingly, while no significant reproducible difference could be observed at the transcript level (Fig. 3*F*; ref. (22)), RBOHD protein accumulation was higher in *cbe1* mutants, while unchanged in *bak1-5* (Figs. 3*G* and S5*A* and S6*A*).To further investigate this phenotype, we analyzed *RBOHD* transcript and protein stability. RNA abundance of *RBOHD* was stable in Col-0 and *cbe1* mutants after treatment with the transcription inhibitor cordycepin (Fig. S5*B*). These results suggest that CBE1 regulates RBOHD translationally or post-translationally, which could thus explain the effect on ROS production and immunity. Moreover, higher elicitor-induced ROS production in *cbe1* mutants was phenocopied by overexpressing *RBOHD* in WT and *bak1-5* (Fig. S6*B*).

### CBE1 colocalizes with ribonucleoprotein complexes

**Figure 4:**
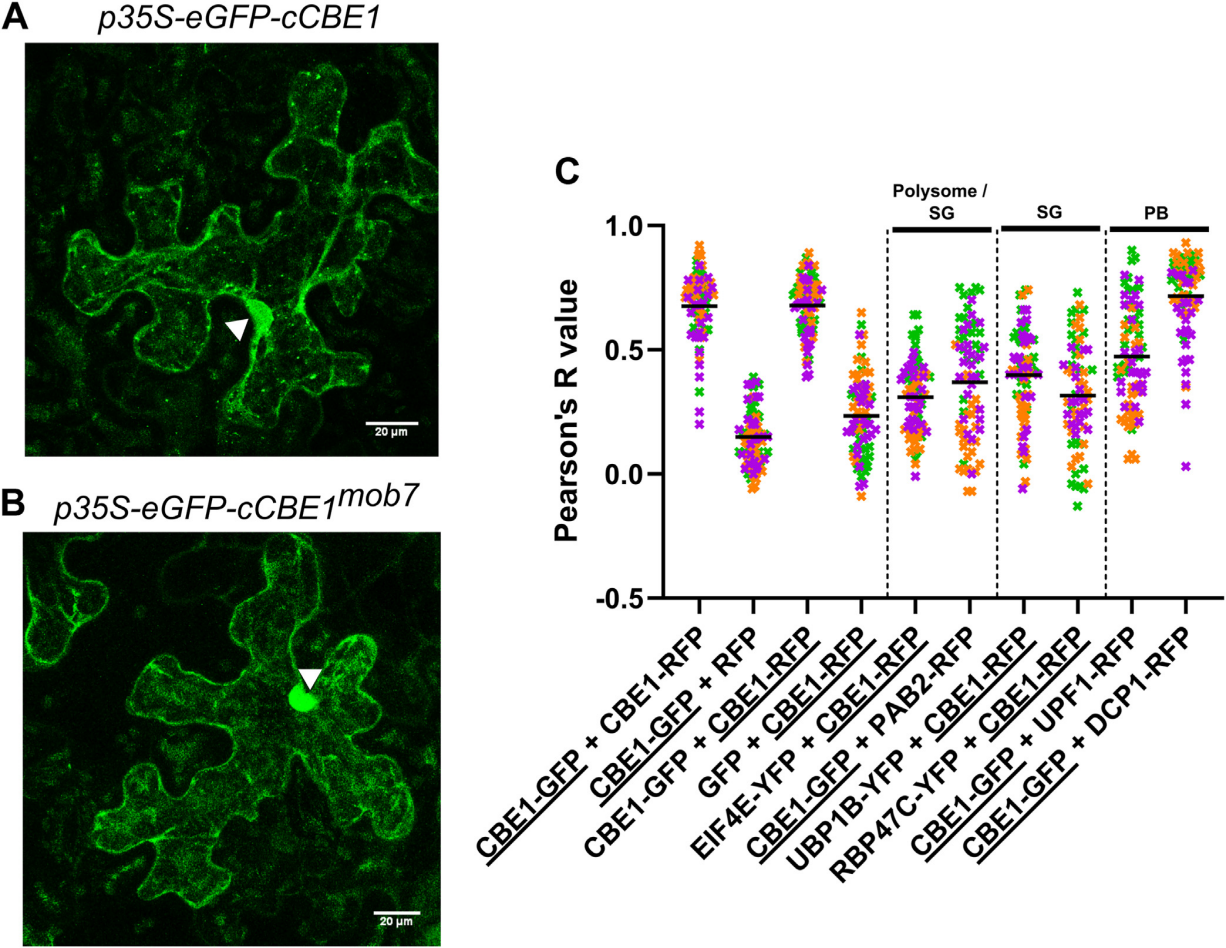
**CBE1 localizes predominantly to processing bodies among ribonucleoprotein complexes**. *A* and *B*, confocal images of CBE1-GFP (*A*) or CBE1^*mob7*^-GFP (*B*) after transient expression in *N. benthamiana*. Each picture is a z-stack projection. The scale bar corresponds to 20 μm. *C*, quantitative colocalization analysis for CBE1 with polysomes/stress granules (SGs), SG-specific and P-bodies (PB) markers after transient co-expression in *N. benthamiana*. The Pearson correlation coefficient (R) was calculated with five ROIs (25 μm^2^) per image (n = 5, images) and the proteins *underlined* refer to the channel used to draw the ROIs. Representative images are shown in Fig. S7. CBE1, conserved binding of eIF4E1.

CBE1 is known to interact with the translation initiation factors eIF4E and eIFiso4E, which localize to ribonucleoprotein complexes associated with the 5′ cap of mRNA transcripts (22). We were therefore interested to investigate the subcellular localization of CBE1. When transiently expressed in *N. benthamiana*, CBE1-GFP displays a nucleocytoplasmic subcellular distribution, additionally localizing to distinct cytoplasmic foci (Fig. 4*A*). Comparatively, while CBE1^*mob7*^-GFP similarly localizes to the cytoplasm and nucleus, localization in cytoplasmic foci was not apparent (Fig. 4*B*). To investigate the localization of CBE1 within cytoplasmic foci, colocalization was measured using Pearson correlation coefficient with different ribonucleoprotein complex markers (31). Active translation is located within polysomes while processing bodies (P-bodies) and stress granules are generally associated with decay and storage of mRNA, respectively (32). To differentiate those different subcomplexes, we used relevant marker proteins. Associated with P-bodies, decapping 1 (DCP1) (33) is a member of the decapping complex, which is responsible for removal of the 5′ cap, while up-frameshift suppressor 1 (34) is a factor of NMD. Although generally associated with active translation within polysomes, the translation initiation factor eIF4E (35) and poly(A) binding protein 2 (36) also localize to stress granules, together with the RNA-binding proteins (RBPs) oligouridylate-binding protein 1b (36) and RNA-binding protein 47C (35). We observed the highest colocalization correlation between CBE1 and DCP1 as well as partial colocalization between CBE1 and up-frameshift suppressor 1 (Figs. 4*C* and S7*A*). To a lesser extent, CBE1 also colocalized with polysome and stress granule markers eIF4E, oligouridylate-binding protein 1b, RNA-binding protein 47C, and poly(A) binding protein 2 (Figs. 4*C* and S7). The localization of CBE1 into these compartments in *N. benthamiana* was not influenced by flg22 treatment (Fig. S7*B*, Videos S1 and S2). This indicates that CBE1 constitutively colocalizes with ribonucleoprotein complexes and suggests a role for CBE1 in P-bodies.

### RBOHD accumulation is affected in mutants of additional translation factors

**Figure 5:**
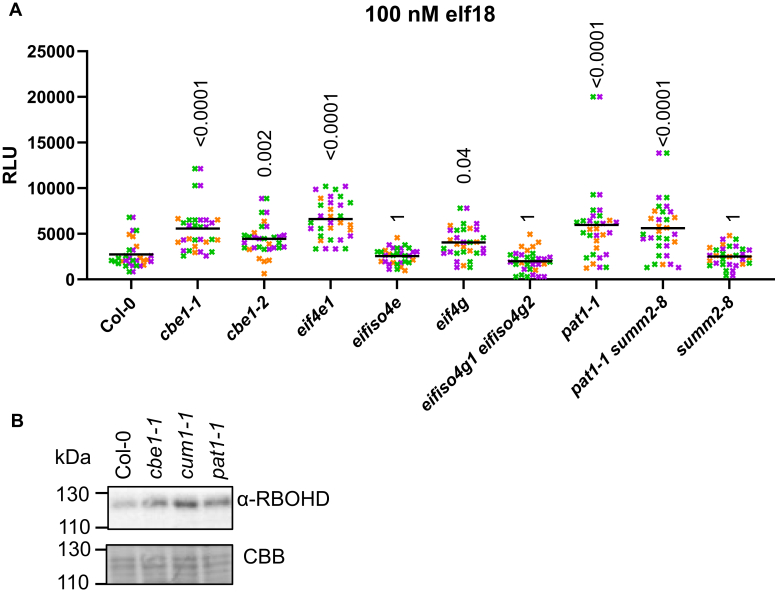
**Translation factor eIF4E and decapping factor PAT1 also play a role in ROS production.***A*, total ROS accumulation measured as RLU over 60 min recording after treatment with 100 nM elf18 on leaf discs from 5-week-old plants: *Horizontal lines* represent the means from three independent experiments (n = 8–12). The *symbol colors* indicate the different experiments. Numbers above symbols are *p*-values from Dunn’s multiple comparison test between the corresponding genotypes and Col-0. *B*, immunoblot analysis of RBOHD (anti-RBOHD) protein accumulations in 5-week-old Arabidopsis leaves from the corresponding genotypes. Coomassie Brilliant Blue (CBB) stain is shown as loading control. Experiment was repeated twice with similar results. RBOHD, respiratory burst oxidase homolog D; RLU, relative light units.

We next tested if RBOHD accumulation and subsequent immune outputs are affected in mutants lacking components of the translation initiation complex (*i.e.*, eIF4E1, eIFiso4E, eIF4G, eIFiso4G1/2) (37), or P-bodies (*i.e.*, PAT1) (38). As PAT1 was shown to be guarded by the nucleotide-binding site leucine-rich repeat receptor suppressor of MKK1 MKK2 2 (SUMM2) (38), the double mutant *pat1-1 summ2-8* was also analyzed together with the single mutants *pat1-1* and *summ2-8*. Similar to *cbe1-1*, *eif4e1* and *pat1* mutants, and to a lesser extent *eif4g*, showed a similar ROS phenotype upon elicitor treatment as observed in *cbe1* (Fig. 5*A*). Accordingly, *eif4e1* and *pat1-1* mutants also displayed increased RBOHD protein levels similar to *cbe1* (Fig. 5*B*), suggesting that RBOHD levels may be regulated by these factors.

## Discussion

Immune signaling relies on tight regulation to allow a proportional and timely response (11, 39). Here, we report that CBE1 contributes to RBOHD protein accumulation and consequently elicitor-induced ROS production and antibacterial immunity. Similarly, mutants of the decapping factor PAT1 and the translation initiation factor eIF4E phenocopy *cbe1*. Overall, this suggests that CBE1, PAT1, and eIF4E regulate RBOHD levels translationally and thereby affect elicitor-induced ROS production. Translational regulation of plant immunity has recently been proposed, as elicitor perception induces global translational reprogramming (40, 41, 42) and remodeling of the cellular RNA-binding proteome (43). Notably, some of these RBPs control transcripts encoding important immune signaling components. For example, alternative splicing targets genes encoding PRRs, kinases, transcription factors, and leucine-rich repeat receptors (44, 45, 46, 47, 48, 49, 50, 51). In addition, the decapping and deadenylation protein complex as well as NMD factors have been shown to regulate stress-responsive transcripts (52, 53, 54, 55, 56, 57). Accordingly, these changes at the level of RBPs and transcripts contribute to plant immune responses against viruses (which depend on host translation) and other pathogens (43, 56, 57).

ROS play an important role for biological processes such as plant development and responses to abiotic and biotic stresses but are also extremely reactive and toxic at high levels, making their regulated production critical to homeostasis (58). Fine-tuning of ROS accumulation happens at different levels in space and time (58), including post-translational modification of NAPDH oxidases. For instance, the most highly expressed NAPDH oxidase, RBOHD, is actively regulated to fine-tune ROS production to permit growth, signaling, and development while avoiding toxicity at high level (58, 59, 60, 61, 62, 63). Recently, post-translational modifications through phosphorylation and ubiquitination of RBOHD were shown to regulate its accumulation during immunity (63). Our work here suggests that CBE1 and other translational regulators represent another layer of regulation of RBOHD protein accumulation; however, the exact underlying mechanistic details remain unknown. Nevertheless, this study further emphasizes the importance of regulating ROS production through modulation of RBOHD abundance. Investigating if CBE1 binds *RBOHD* transcripts directly or binds other transcripts whose products regulate RBOHD levels will be important to further understand the role of CBE1. To determine if this is part of a regulated attenuation mechanism, it will also be necessary to determine if RBOHD is under immune-induced translational control. Interestingly, recent results demonstrated that during immune signaling, *RBOHD* transcripts increased in the set of ribosome-loaded mRNAs (64). However, the role of CBE1 in that process is still unknown, and expressing CBE1 in plants and bacteria has proven challenging (22). Accordingly, we failed to generate stable Arabidopsis transgenic lines expressing epitope-tagged CBE1 despite multiple attempts (Table S2). This highlights the importance of generating novel tools to answer these questions in future studies.

Based on previous work showing the association between CBE1 and eIF4E1 (22), as well as the colocalization and mutant analysis presented here, we suggest that CBE1 might work together with decapping factor DCP1 and translation initiation factor eIF4E1 to regulate RBOHD protein level and consequently elicitor-induced ROS production and immunity. We found that mutants lacking initiation factor *eif4e* showed similar enhanced sensitivity to elf18 as *cbe1*, whereas mutants in other initiation factors (*eif4iso4e* and *eifiso4g1 eifiso4g2*) were indistinguishable from WT. These results are in accordance with the specificities of the different eIF isoforms, which bind the 5′ mRNA cap with a range of affinities (65, 66). We also observed enhanced elf18-induced ROS and RBOHD accumulation in *pat1-1*, which is surprising as eIF4E1 and PAT1 are predicted to function antagonistically. Indeed, eIF4E initiates recruitment of the initiation complex and subsequent recruitment of ribosomes, whereas PAT1 contributes to decapping, which initiates 5′-3′ decay by exoribonucleases (38). In addition, CBE1 seems to localize predominantly to P-bodies, which are generally associated with mRNA decay (67). Interestingly, the number of P-bodies increases when Arabidopsis is treated with flg22 (38, 56), suggesting a link between P-body-mediated mRNA stability and immunity. Yet, we could not observe any increase in CBE1 levels or colocalization with P-bodies in *N. benthamiana* upon flg22 treatment, which could however be due to heterologous overexpression. Given that CBE1 is a plant-specific and nonessential protein, it has been proposed to regulate targeted transcripts in a context-dependent manner (22), which could conceivably provide a fine-tuning mechanism to regulate gene expression. Further work is needed to understand how CBE1 functions in translation initiation and/or mRNA decay.

## Experimental procedures

### Plant materials and growth conditions

*A. thaliana* plants were grown on soil as one to four plants per pot (7 × 7 cm) in controlled environment rooms maintained at 20 °C with a 10-h photoperiod (150 μmol m^−2.^s^−1^), and 60% humidity, or as seedlings on sterile Murashige and Skoog (MS) media supplemented with vitamins and 1% (w/v) sucrose (Duchefa) with a 16-h photoperiod (120 μmol m^−2.^s^−1^). Assays using soil-grown plants were performed at 4 to 6 weeks postgermination, before the reproductive transition. Assays using plate-grown seedlings were performed at 2 weeks postgermination. *A. thaliana* ecotype Columbia-0 (Col-0) was used as a wildtype control for all plant assays and was the background for all mutants used in this study, except otherwise stated. The *bak1-5 mob7* mutant was purified by one backcross to *bak1-5*. The single *mob7* mutant was obtained by crossing *bak1-5 mob7* to Col-0. Knockdown alleles *cbe1-2* (*AT4G01290*; SALK_038452) and *cbe1-3* (*AT4G01290*; GK_150_H09) and wildtype alleles denoted *CBE1-2* and *CBE1-3* were derived by segregation of SALK_038452 and GK_150_H09, respectively, and were obtained through the Nottingham Arabidopsis Stock Centre (NASC). Ecotype Landsberg *erecta* (L*er-*0) and *rbohD* (SLAT line) (68) and *bak1-5* (EMS mutant) (23) were previously described and already available in our seed collection. Genotypes *cbe1-1* (WiscDsLoxHs188_10F) (22), *eif4e1* (*cum1*-*1*; nonsense mutation in *EIF4E1*) (69), *eif4g* (SALK_80031) (22), *eifiso4e* (SLAT line) (70), and double mutant *eifiso4g1 eifiso4g2* (SALK_009905; SALK_076633) (71) were obtained from Karen Browning. Genotypes *pat1-1* (SALK_040660), *summ2-8* (SAIL_1152A06), and *pat1-1 summ2-8* (38) were obtained from Morten Petersen.

*N. benthamiana* plants were grown on soil as one plant per pot (8 × 8 cm) at 25 °C during the day with 16 h light (120 μmol m^−2.^s^−1^) and at 22 °C during the night (8 h). Relative humidity was maintained at 60%.

### Map-based cloning and whole-genome sequencing

The *bak1-5 mob7* mutant (in Col-0) was crossed to L*er*-0. Fifty-six F_2_ segregants were genotyped for *bak1-5* using a dCAPS marker (Table S1). Homozygous *bak1-5* segregants were phenotyped for elf18-induced ROS production as for *mob7*. Linkage analysis was performed using an array of genome-wide markers designed in-house or by the Arabidopsis Mapping Platform (Table S1) (72). For whole-genome sequencing, 440 F_2_ plants from the cross *bak1-5 mob7* with *bak1-5* were scored for chitin-induced ROS production. One hundred thirty-three plants showed moderately increased and 93 plants highly increased ROS production. Out of these 93 plants, 70 were tested in the F_3_ generation, and only 15 showed a confirmed phenotype to restore Atpep1-induced seedling growth inhibition in three experiments. Thirty seedlings from each of the positive F_3_ parents were bulked and ground to a fine powder in liquid nitrogen and gDNA extracted. Ground tissues were equilibrated in buffer containing 50 mM Tris-HCl (pH 8.0), 200 mM NaCl, 2 mM EDTA for 30 min at 37 °C with occasional mixing, and a further 20 min at 37 °C with 0.2 mg/ml RNase. Roughly 10 ng of genomic DNA was then extracted using a standard chloroform/phenol method and resuspended in TE buffer (10 mM Tris HCl pH 7.5; 1 mM EDTA pH 8). Prepared gDNA of pooled *bak1-5 mob7* F_3_ segregants, as well as *bak1-5* as a reference (12), was submitted to The Beijing Genomics Institute (Hong Kong) for Illumina-adapted library preparation and paired-end sequencing using the High-Seq 2000 platform. The average coverage from Illumina sequencing of *bak1-5 mob7* over the nuclear chromosomes was 15.79. Paired-end reads were aligned to the TAIR10 reference assembly using BWA v 0.6.1 with default settings (73). BAM files were generated using SAMTools v 0.1.8 (73), and single-nucleotide polymorphisms (SNPs) were called using the mpileup command. High-quality SNPs were obtained using the following filters: (a) Reads with mapping quality less than 20 were ignored; (b) SNP position had a minimum coverage of six and a maximum of 250; (c) the reference base must be known; and (d) SNPs were present in *bak1-5 mob7* but not in the *bak1-5* control. The resulting pileup files contained a list of SNPs and their genomic positions. SNPs unique to *bak1-5 mob7* and not present in the *bak1-5* control were identified. SNPs passing filters were analyzed on CandiSNP (74). Relevant SNPs were confirmed in the original *bak1-5 mob7* mutant and backcrossed lines by Sanger sequencing of PCR amplicons.

### Elicitors

The following elicitors were used in this study: chitin (Yaizu Suisankagaku Industry), flg22 peptide (QRLSTGSRINSAKDDAAGLQIA) (75), elf18 peptide (ac-SKEKFERTKPHVNVGTIG) (76), and Atpep1 peptide (ATKVKAKQRGKEKVSSGRPGQHN) (77). All peptides were synthesized by SciLight-peptide (China) with purity above 95% and dissolved in sterile distilled water.

### Oxidative burst assay

ROS production was measured as previously described (23). For the assay, either adult plants (4- to 6-week-old plants) or seedlings (2-week-old) were used. For adult plants, leaf discs (4-mm diameter) were collected using a biopsy punch and floated overnight on distilled, deionized water in a white 96-well plate to recover from wounding. For ROS assays on whole seedlings, seedlings were grown on MS agar plates for 5 days before being transferred to MS liquid medium in transparent 96-well plates. After 8 days, seedlings were transferred to a white 96-well plate and allowed to recover overnight in sterile water. The water was then removed and replaced with elicitor solution containing 17 μg/ml luminol (Sigma-Aldrich), 100 μg/ml horseradish peroxidase (Sigma-Aldrich), and the indicated elicitor concentration. For seedlings, the hyperactive luminol derivative 0.5 μM L-012 (Fujifilm Wako Chemicals) was used instead of luminol. Luminescence was recorded over a 40- to 60-min period using a charge-coupled device camera (Photek Ltd).

### Seedling growth inhibition assay

Seedling growth inhibition was performed as previously described (23). Sterilized and stratified seeds were sown on MS media and grown in controlled environment rooms with 16/8 h day/night cycle and constant temperature of 22 °C. Five-day-old seedlings were transferred into liquid MS with or without the indicated amount of elicitor. 10 to 12 days later, individual seedlings were gently dry-blotted and weighed using a precision scale (Sartorius).

### MAP kinase phosphorylation assay

Phosphorylation of MAPKs was measured as previously described (78). Leaf discs (4-mm diameter) from adult plants (4- to 6-week-old plants) were cut in the evening and left overnight on the bench, floating in 6-well plates on distilled, deionized water. In the morning, the elicitor peptide was added to the desired concentration, and tissue was blotted dry and flash-frozen in liquid nitrogen for protein extraction at the indicated time points. MAPK phosphorylation was detected by Western blot using an antibody specific to the active phosphorylated form of the proteins (phospho-p44/42 MAPK). Fifteen leaf discs were used per condition.

### Bacterial spray inoculation

Spray inoculations were performed as previously described (79). *P. syringae* pv. *tomato* (*Pto*) DC3000 wildtype and *COR*^*-*^ (defective in production of the phytotoxin coronatine) strains (80) were grown in overnight culture in King’s B medium supplemented with 50 μg/ml rifampicin, 50 μg/ml kanamycin, and 100 μg/ml spectinomycin and incubated at 28 °C. Cells were harvested by centrifugation and pellets resuspended in 10 mM MgCl_2_ to an *A*_600_ of 0.2, corresponding to 1 × 10^8^ colony forming units (CFU)/ml. Immediately before spraying, Silwet L-77 (Sigma Aldrich) was added to a final concentration of 0.04% (v/v). Four-to five-week-old plants were uniformly sprayed with the suspension and covered with a clear plastic lid for 3 days. Three leaf discs (4-mm diameter) were taken using a biopsy puncher from three respective leaves of one plant and ground in collection microtubes, containing one glass bead (3-mm diameter) and 200 μl water, using a 2010 Geno/Grinder (SPEX) at 1500 rpm for 1.5 min. Ten microliters of serial dilutions from the extracts were plated on LB agar medium containing antibiotics and 25 μg/ml nystatin (Melford). Colonies were counted after incubation at 28 °C for 1.5 to 2 days.

### Molecular cloning

Gateway-compatible fragments were amplified using Phusion Taq polymerase (New England Biolabs) from either Col-0 genomic DNA (*gCBE1*) containing 2.5 kb of the promoter sequence upstream of the translational start codon or from Col-0 complementary DNA (*cCBE1*) or from *mob7* cDNA (*cCBE1*^*mob7*^) and with or without the endogenous stop codon. Gateway ‘attB’ flanked PCR products were cloned into pDONR201 using BP Clonase II (Invitrogen), and recombination was performed using LR Clonase II (Invitrogen) into the corresponding destination vector (pK7WGF2.0, pK7FWG2.0, pGWB604, pUBC-GFP-Dest, pB7WGR2.0) (81, 82, 83). All clones were verified by Sanger sequencing.

### Transient expression in *N. benthamiana*

*N. benthamiana* plants were used for transient transformation at 4- to 5-weeks postgermination. *Agrobacterium tumefaciens* GV3101 overnight cultures grown at 28 °C in LB were harvested by centrifugation at 2500*g* and resuspended in buffer containing 10 mM MgCl_2_ and 10 mM MES for 3 h at room temperature. *A. tumefaciens*-mediated transient transformation of *N. benthamiana* was performed by infiltrating leaves with *A*_600_ = 0.2 of each construct together with the viral suppressor P19 (84) in a 1:1 (or 1:1:1) ratio. Samples were collected 2 to 3 days after infiltration.

### Stable transformation of Arabidopsis

Transgenic Arabidopsis plants were generated using floral dip method (85). Briefly, flowering plants were dipped into a suspension culture of *A. tumefaciens* GV3101 carrying the indicated plasmid. Plants carrying a T-DNA insertion event were selected either on MS medium containing the appropriate selection or as soil-grown seedlings by spray application of Basta (Bayer Crop Science). T_1_ seedlings resistant to selective marker on MS plate were transferred to soil to produce the next generation. T_2_ resistance was monitored to find single insertion lines, while T_3_ resistance was screened for homozygous mutants and expression of tagged lines verified by Western blot.

### Confocal microscopy

*N. benthamiana* leaf discs (4-mm diameter) transiently overexpressing the indicated proteins were sampled at 2 to 3 dpi with water as the imaging medium. For elicitor treatment in *N. benthamiana*, leaf discs were harvested 3 dpi and incubated overnight in petri dishes containing water. The next day, leaf discs were transferred to microscopic slides containing 1 μM flg22 or water. Live-cell imaging employed a laser-scanning Leica SP5 Confocal Microscope (Leica Microsystems) and 63x (glycerol immersion) objective. GFP was excited at 488 nm and emission detected between 496 and 536 nm (shown in green). YFP was excited at 514 nm and detected between 524 and 551 nm (shown in yellow). RFP derivatives (mRFP, mCherry, tag-RFP) were excited at 561 nm and detected between 571 and 635 nm (shown in magenta). Colocalization was performed using sequential channel analysis by calculating Pearson’s coefficient (31, 86) using the Coloc 2 plugin of ImageJ. Image analysis was performed with Fiji (87).

### Immunoblot analysis

Plant tissues were ground in liquid nitrogen, and protein was extracted using a buffer containing 50 mM Tris-HCl, pH 7.2; 150 mM NaCl; 1 mM EDTA; 5% glycerol; 5 mM DTT; and 1% (v/v) Protease Inhibitor Cocktail (P9599, Sigma-Aldrich), boiled for 10 min, and debris removed by centrifugation for 2 min at 12,000*g*. Protein samples were separated by 8% or 12% (pMAPK) sodium dodecylsulfate polyacrylamide gel electrophoresis and blotted onto a polyvinylidene difluoride membrane (Thermo Fisher Scientific). Immunoblotting was performed with antibodies diluted in blocking solution (5% nonfat milk in TBS with 0.1% [v/v] Tween-20) at the following titers: anti-GFP (1:5000; Santa Cruz; sc-9996); anti-RFP-HRP (1:5000; Abcam; ab34767); anti-mouse IgG-HRP (1:15,000; Sigma Aldrich; A0168); anti-rabbit IgG-HRP (1:10,000; Sigma Aldrich; A6154); anti-RBOHD (1:1000; Agrisera; AS15 2962); and anti-phospho-p42/p44-erk (1:1000; Cell Signalling Tech; #9101). Blots were developed with Pierce ECL Pico Western Blotting substrate (Thermo Fisher Scientific). Protein loading was verified by staining the blotted membrane with Coomassie Brilliant Blue G-250.

### RNA extraction and qPCR analysis

Total RNA was extracted using Trizol reagent (Invitrogen) according to the manufacturer’s instructions. RNA samples were treated with Turbo DNA-free DNase (Ambion) according to the manufacturer’s instructions. RNA was quantified with a Nanodrop spectrophotometer (Thermo Fisher Scientific). cDNA was synthesized from RNA using RevertAid (Thermo Fisher Scientific) according to the manufacturer’s instructions. Quantitative PCR was conducted following the MIQE guidelines (88) using a 7500 Real-Time PCR System (Applied Biosystems) and PowerUp SYBR Green Master Mix (Applied Biosystems) with cDNA diluted 1:20. The 2^−ΔCt^ method was used for the calculation of relative expression.

### RNA stability assay

RNA stability was measured as previously described (89). Briefly, three leaf discs from different plants (5-week-old) were collected in 24-well plate with 0.5 ml sterile water. The next day cordycepin (Chengdu Biopurify Phytochemicals) was added to a final concentration of 0.6 mM and discs were sampled at 0, 30, 60, 90, or 120 min, blotted dry, and flash frozen.

### Statistical analysis

Statistical analysis was performed using R (4.1.2) and Rstudio (2021.09.1) or GraphPad Prism (9.3). Based on Gaussian distribution, parametric or nonparametric tests were chosen and when n ≥ 30, normal distribution was assumed. Prior to multiple comparisons, ANOVA or Kruskal–Wallis test were performed to assess differences across groups. For multiple comparisons, Dunnett’s and Dunn’s tests were favored to compare multiple groups to one control group. Tests were realized on the overall set of replicates, and replicates were included only when positive and negative controls showed the expected results.

## Data availability

All data are contained within the manuscript.

## Supporting information

This article contains supporting information.

## Conflict of interest

The authors declare no conflict of interest with the contents of this article.
