# Supplementary material for: Arabidopsis translation initiation factor binding protein CBE1 negatively regulates accumulation of the NADPH oxidase respiratory burst oxidase homolog D
Source: J Biol Chem. 2023 Jul 7;299(8):105018. doi: 10.1016/j.jbc.2023.105018 (PMC10432800; doi:10.1016/j.jbc.2023.105018)
Supplement: Supporting Information [file mmc3.pdf]

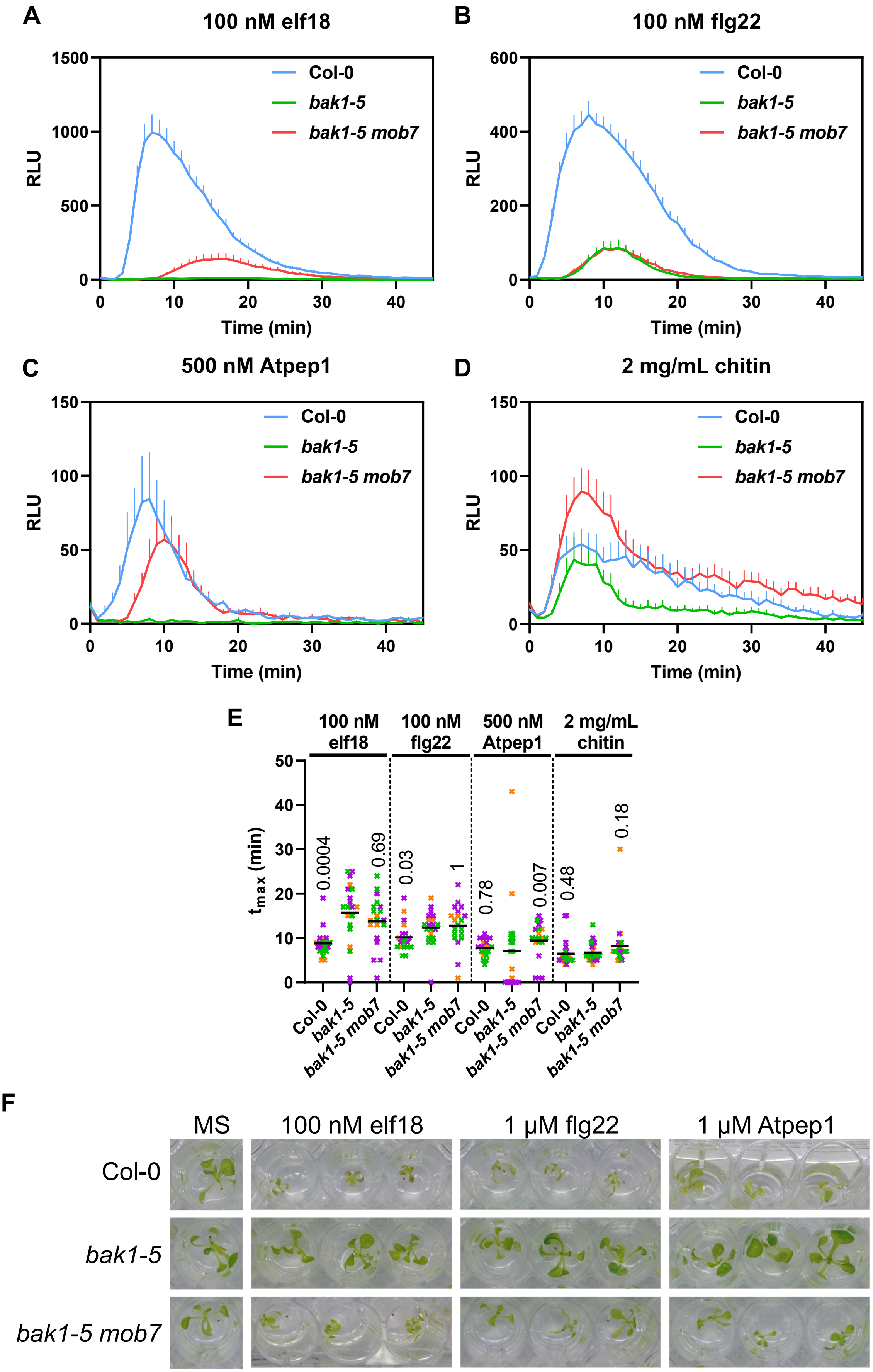

Figure S1. *mob7* restores immune signaling in *bak1-5*.
(A-D) ROS burst kinetic measured as relative light units (RLU), in leaf discs following treatment with 100 nM elf18 (A) or 100 nM flg22 (B) or 500 nM Atpep1 (C) or 2 mg/mL chitin (D). Values are means + standard errors (n=8). (E) T_{max} describes the time it takes for the ROS to peak upon treatment with corresponding elicitors over 60 min recording. Horizontal lines represent the means from 3 independent experiments (n=4-8). The symbol colors indicate the different experiments. Numbers above symbols are p-values from Dunn's multiple comparison test between corresponding genotype and *bak1-5*. (F) Images of 14-day-old seedlings grown in MS media or MS media containing 100 nM elf18, 1 µM flg22 or 1 µM Atpep1.

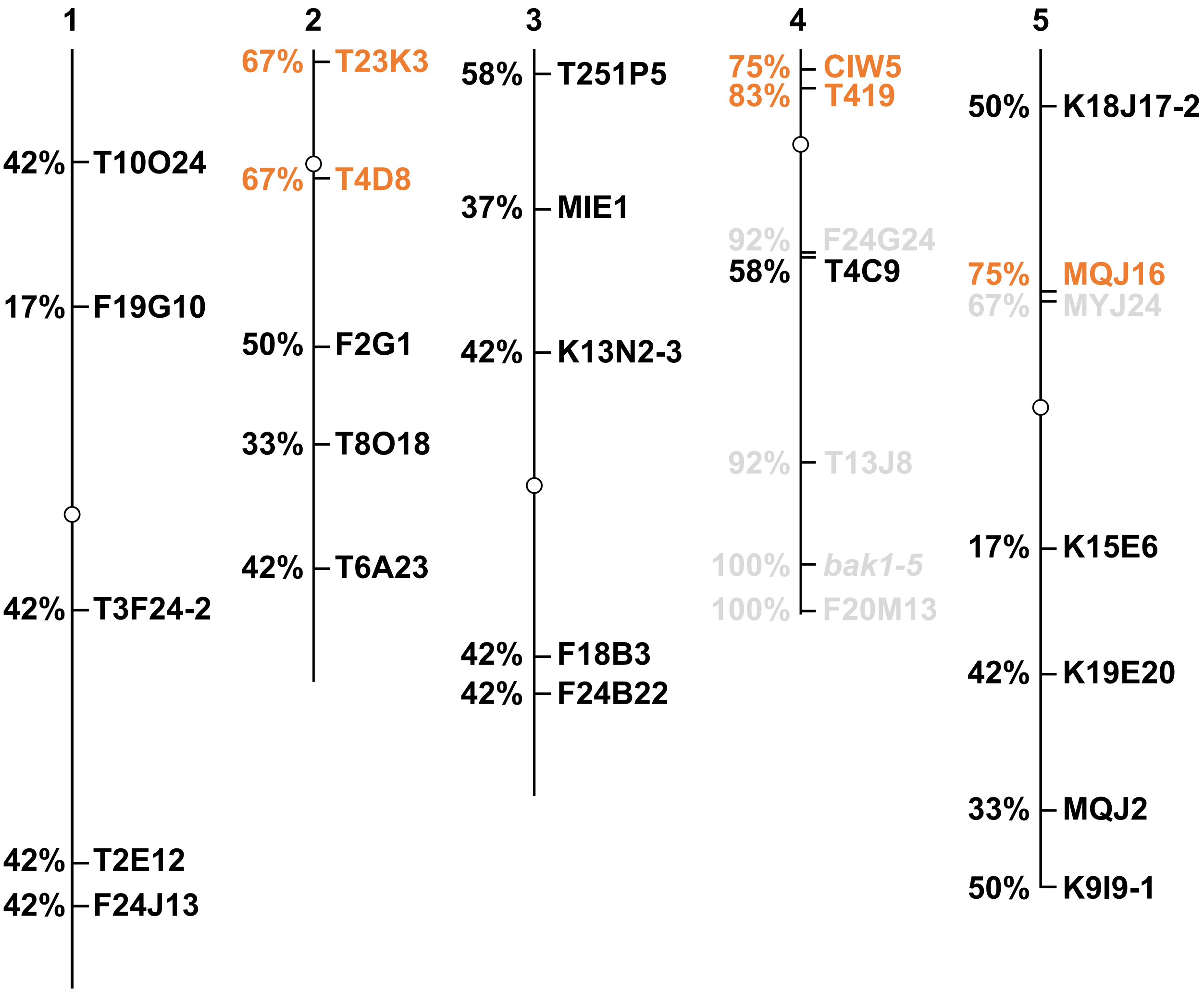

Figure S2. Map-based cloning of *bak1-5 mob7*.
Physical linkage map constructed using the F_2 population from *bak1-5 mob7* x Ler-0. The percentage represent the percentage of Col-0 alleles contributed by *bak1-5 mob7*. Plants were screened based on ROS production upon treatment with 100 nM elf18. Markers in grey are markers for which an increase of Col-0 alleles was also observed in plants with low elicitor-induced ROS production, thereby removed from further analysis. Markers in orange show linkage statistically higher than 50%. Circles represent centromeres. Significance was determined by χ^2 test.

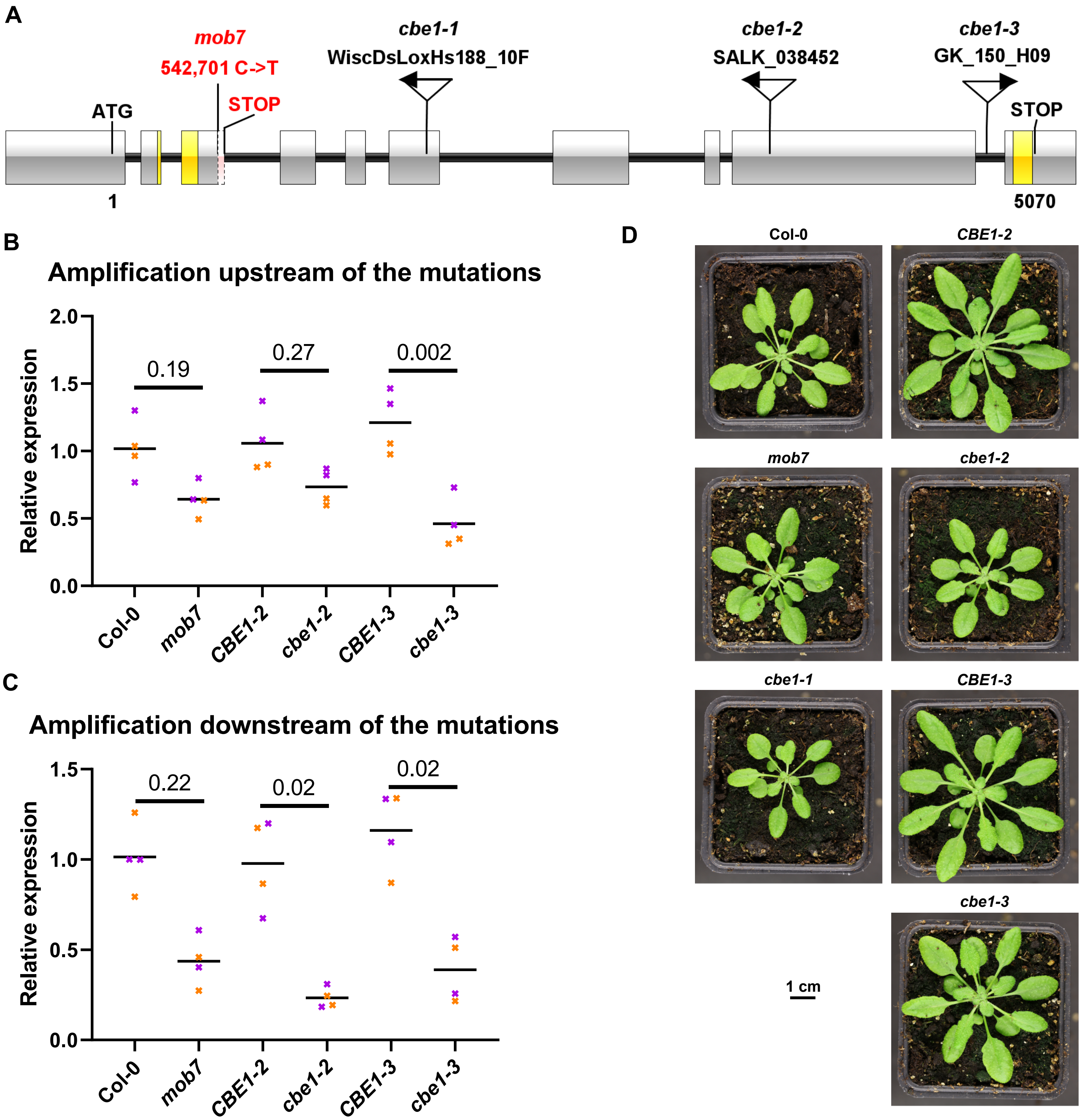

Figure S3. Characterization of *cbe1* alleles.
(A) Gene structure of *AT4G01290*. Exons are shown as grey boxes. T-DNA insertions are indicated with the respective name above and arrows indicate the orientation of the T-DNAs. EMS-induced *mob7* mutation with respective position and early stop codon are indicated in red. Fragments amplified by quantitative reverse-transcription polymerase chain reaction (RT-qPCR) are indicated in yellow. (B,C) RT-qPCR of *AT4G01290* upstream of the T-DNA insertions/*mob7* mutation (B) or downstream of the insertions/mutation (C). (B,C) Expression values relative to the *U-BOX* housekeeping gene are shown. *CBE1-2* and *CBE1-3* are *CBE1* wildtype segregants from the *cbe1-2* and *cbe1-3* lines, respectively. Horizontal lines represent the means from 2 independent experiments (n=2) (B,C) The symbol colors indicate the different experiments. Numbers above horizontal lines are p-values from Dunn's multiple comparison test between genotypes under the lines. (D) Rosette morphology of 5-week-old plants of the corresponding genotype.

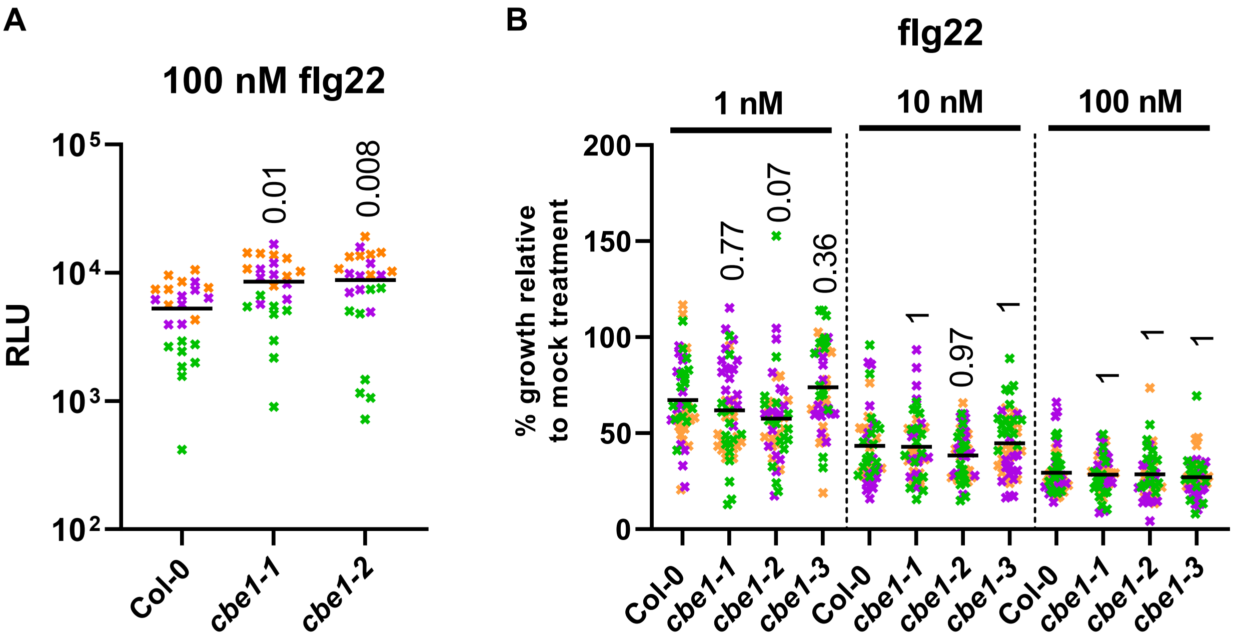

Figure S4. CBE1 negatively regulates elicitor-induced ROS production.
(A) Total ROS accumulation measured as RLU over 60 min recording after treatment with 100 nM flg22 on leaf discs from 5-week-old plants. Horizontal lines represent the means from 3 independent experiments (n=8). (B) Growth inhibition represented as percentage of fresh weight in response to 1, 10 or 100 nM flg22 relative to mock treated seedlings. Horizontal lines represent the means from 3 independent experiments (n=16). Numbers above symbols are p-values from (A,B) Dunnett's or (C) Dunn's multiple comparison test between corresponding genotype and *bak1-5*.

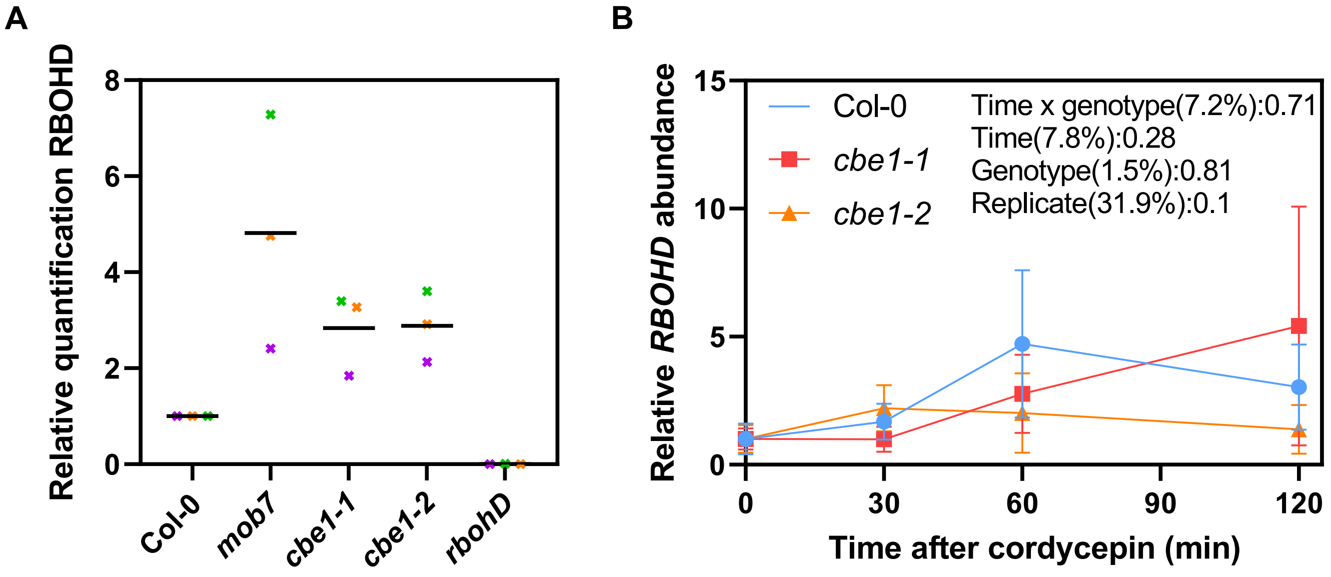

Figure S5. RBOHD accumulation in *CBE1* mutants is not related to changes in transcript.
(A) Quantification of immunoblots of RBOHD (anti-RBOHD) relative to the signal detected after Coomassie Brilliant Blue (CBB) stain at the corresponding size in 5-week-old Arabidopsis leaves from the corresponding genotypes. Values are normalized to Col-0. (B) RNA stability measurement in leaf discs of 5-week-old Arabidopsis leaves after inhibition of transcription by cordycepin treatment of *RBOHD*. *ACTIN* served as an internal control. Results of ANOVA test are shown as p-value and variation within parentheses for each factors and interaction.

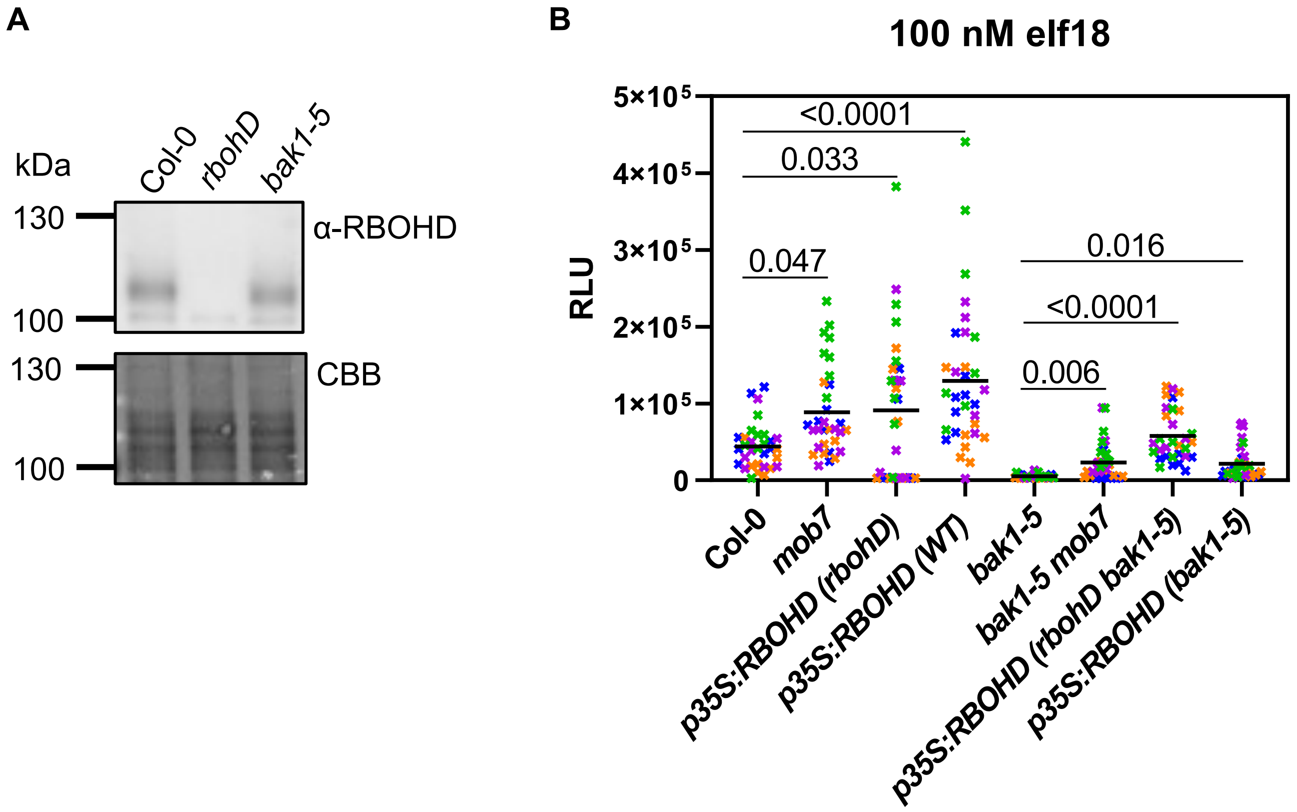

Figure S6. Overexpression of *RBOHD* phenocopies *mob7*.

(A) Immunoblot analysis of RBOHD (anti-RBOHD) protein accumulations in 5-week-old Arabidopsis leaves from the corresponding genotypes. Coomassie Brilliant Blue (CBB) stain is shown as loading control. Experiment was repeated twice with similar results. (B) Total ROS accumulation measured as RLU over 60 min recording after treatment with 100 nM elf18 on leaf discs from 5-week-old plants: Horizontal lines represent the means from 4 independent experiments (n=8). Numbers above horizontal bars are p-values from Dunnett's multiple comparison test between corresponding genotypes. For *RBOHD* transgenics, the genotype background is written between parentheses.

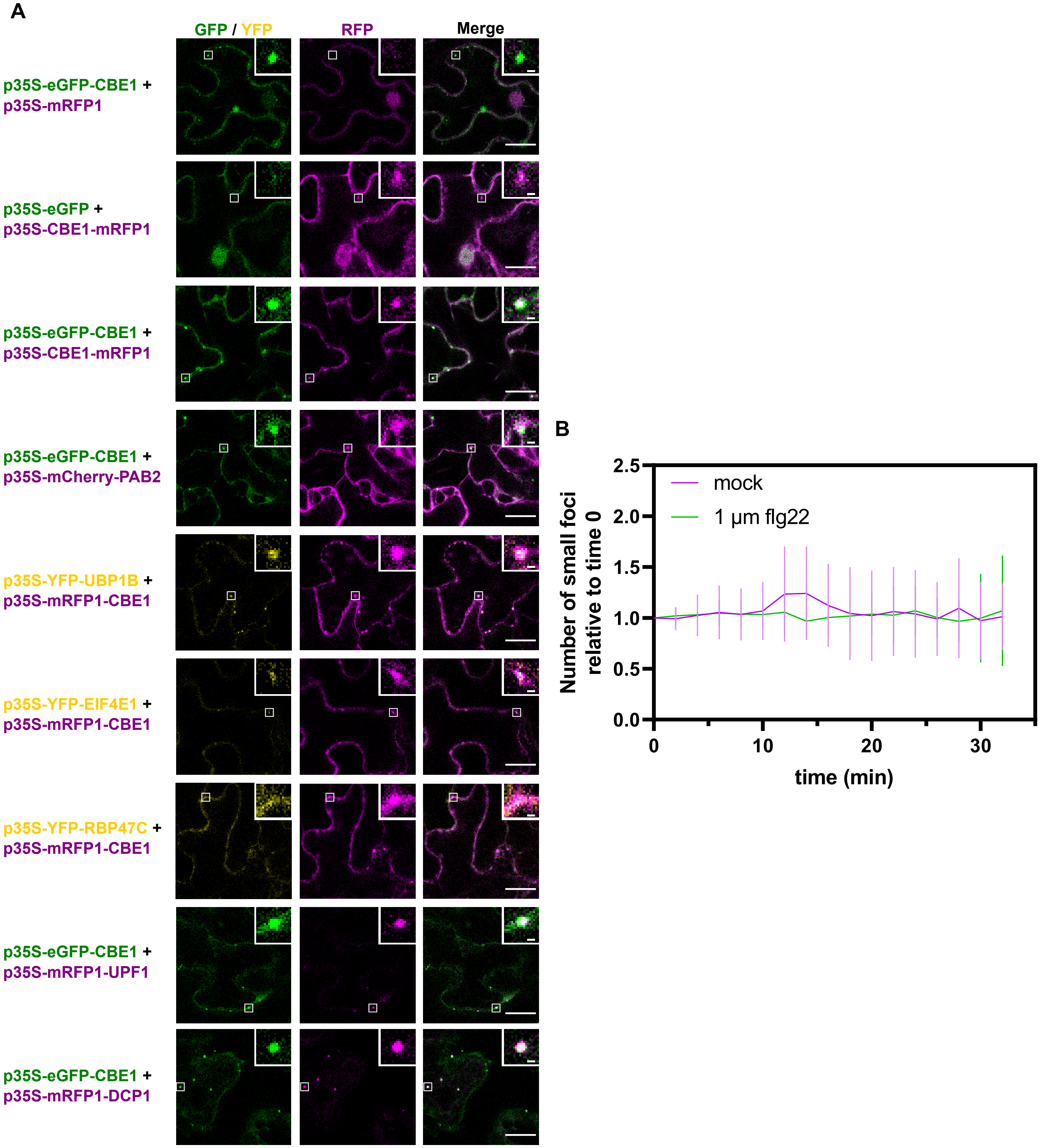

Figure S7. CBE1 localizes predominantly to processing bodies among ribonucleoprotein complexes.
(A) Confocal images of green, yellow and red fluorescent proteins. The proteins were transiently co-expressed in *N. benthamiana*. Merged pictures show overlay of GFP/YFP and RFP. The scale bar corresponds to 20 µm. An ROI of 25 µm^2 is shown by white square and zoomed in on the top right of the images with a scale bar of 1 µm. P-bodies markers: UPF1, DCP1; polysomes/stress granule markers: PAB2, EIF4E1; stress granule markers: UBP1B, RBP47C. (B) Quantification of the number of small foci per z-stack image with an average size of 1.44 µm^2 over 32 min after treatment with 1 µM flg22 or water (mock). (A,B) The construct *p35S-eGFP-CBE1* was transiently co-expressed in *N. benthamiana*. Confocal microscopy on leaf discs was conducted 3 days post-infiltration.

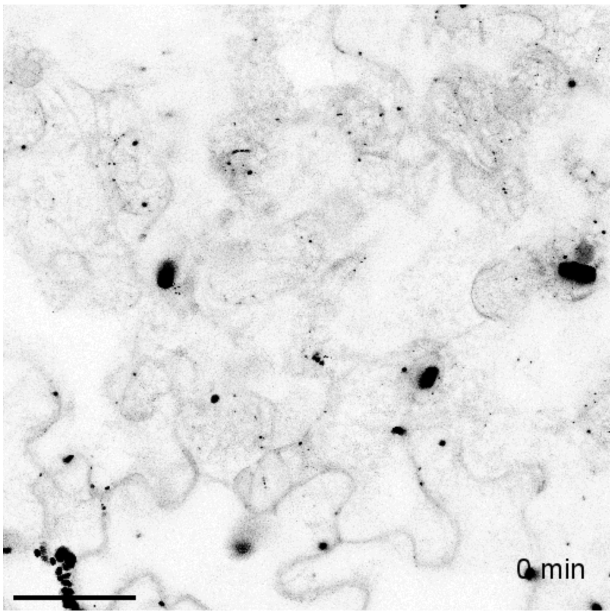

Video S1. CBE1 localization over time (mock)
Confocal images of *p35S-eGFP-CBE1* transiently co-expressed in *N. benthamiana*. The video represent z-stack images taken every 2 minutes over 32 min after treatment with water (mock). The colors were reversed for clarity. The scale bar corresponds to 50 μm.

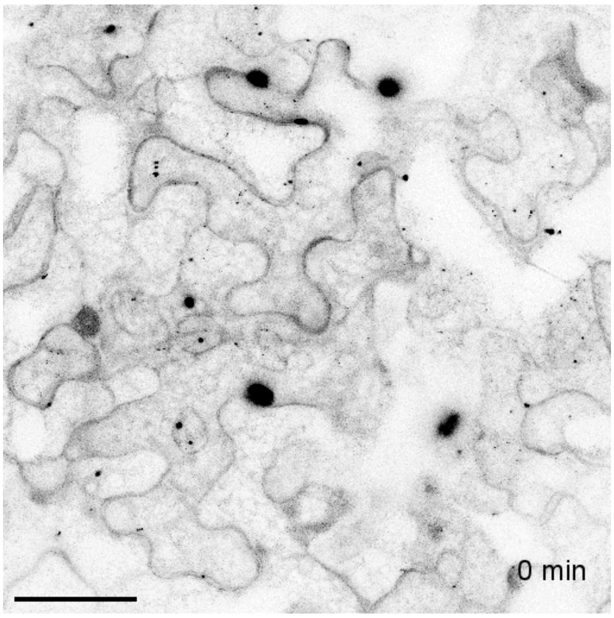

Video S2. CBE1 localization over time (flg22)
Confocal images of *p35S-eGFP-CBE1* transiently co-expressed in *N. benthamiana*. The video represent z-stack images taken every 2 minutes over 32 min after treatment with 1 μm flg22. The colors were reversed for clarity. The scale bar corresponds to 50 μm.

Table S1. CBE1 localizes predominantly to processing bodies among ribonucleoprotein complexes.

Name	Sequence (5'-3')	Purpose	Locus
T10O24.F	ATCAAAACTACGTCGTTTTA	Map-based cloning	
T10O24.R	TTCAAAATCAATCGAACATA	Map-based cloning	
F19G10.F	ATGTCACCGTGAACGACATC	Map-based cloning	
F19G10.R	TGCGAGTTAAGACCTAGGAG	Map-based cloning	
T3F24-2.F	TCCACACACGCAACTTCATGGCAT	Map-based cloning	
T3F24-2.R	TTACTTAGGTGACACGTGTGATGT	Map-based cloning	
T2E12.F	CGACTAGCCAGTCCGATACA	Map-based cloning	
T2E12.R	CGTTTTGGGAGCCACGTTTC	Map-based cloning	
F24J13-2.F	CTTGTAAAACCTCGATATTATCTC	Map-based cloning	
F24J13-2.R	ACTAAGATACTAGTAGGCTCGGCT	Map-based cloning	
T23K3.F	CGTGTTTACCGGGTCGGA	Map-based cloning	
T23K3.R	AAAACCCTTGAAGAATACG	Map-based cloning	
T4D8.F	ATTAACCCCAATGATGCTGA	Map-based cloning	
T4D8.R	AGCGGATAGATAATGGTCAA	Map-based cloning	
F2G1.F	CGTCGTCGGAAGTTTCAGAG	Map-based cloning	
F2G1.R	GAATAAGAAGAACACATGCGTC	Map-based cloning	
T8O18.F	GATATGGATGTAACGACCCAA	Map-based cloning	
T8O18.R	CAGCTTCGAGTGGATTCTAC	Map-based cloning	
T6A23.F	ATGTCCAAATTGACCAACCG	Map-based cloning	
T6A23.R	CAAAATAAACACCCCCAACT	Map-based cloning	
T251P5.F	CATCCGAATGCCATTGTTC	Map-based cloning	
T251P5.R	AGCTGCTTCCTTATAGCGTCC	Map-based cloning	
MIE1.F	CTAAGTTCTTCCACCATCTG	Map-based cloning	
MIE1.R	CAAGGAGCATCTAGCCAGAG	Map-based cloning	
K13N2-3.F	ATTAAATCTAAAATCGAGTGATT	Map-based cloning	
K13N2-3.R	AACAAACATTACTCGGTATCCAGT	Map-based cloning	
F18B3.F	GTTCATTAAACTTGCGTGTGT	Map-based cloning	
F18B3.R	TACGGTCAGATTGAGTGATTC	Map-based cloning	
F24B22.F	CTGGGAACAAAGGTGTCATC	Map-based cloning	
F24B22.R	CAAGGTCTCCAGAACACAAAC	Map-based cloning	
CIW5.F	GGTTAAAAATTAGGGTTACGA	Map-based cloning	
CIW5.R	AGATTTACGTGGAAGCAAT	Map-based cloning	
T419.F	TTATAGCAAACGTACAAGTC	Map-based cloning	
T419.R	CTGCATACACGTCGTCTC	Map-based cloning	
F24G24.F	GCCAAACCCAAAATTGTAAAAC	Map-based cloning	
F24G24.R	TAGAGGGAACAATCGGATGC	Map-based cloning	
T4C9.F	CAAAGGTTTCGTGTCGGAGC	Map-based cloning	
T4C9.R	CGTTGACGGGATACTCGGTG	Map-based cloning	
T13J8.F	ATGTTCCCAGGCTCCTTCCA	Map-based cloning	
T13J8.R	GAGATGTGGGACAAGTGACC	Map-based cloning	
F20M13.F	TCTCGTAAGCAAATCAACGAATAG	Map-based cloning	
F20M13.R	AAGATGCGTGCGTTGATGGACCAA	Map-based cloning	
K18J17-2.F	GGTCCGAATCTAAACTCGGTTAAT	Map-based cloning	
K18J17-2.R	AGTGTTCGAGCAATAAGAGTGATT	Map-based cloning	
MQJ16.F	TAGTGAAACCTTTCTCAGAT	Map-based cloning	
MQJ16.R	TTATGTTTTCTTCAATCAGTT	Map-based cloning	
MYJ24.F	CTAATCCCAAGCTGAATCAC	Map-based cloning	
MYJ24.R	TGACAGAGAATCCGACTGTG	Map-based cloning	
K15E6.F	GGCTGCTTCACTGAGTTG	Map-based cloning	
K15E6.R	AAAAGCCCATTTAAAACG	Map-based cloning	
K19E20.F	GACAAGAACCACATGAGAGC	Map-based cloning	
K19E20.R	GTTATGTGTACACTTCAGGTC	Map-based cloning	
MQJ2.F	ATTCTCCGTAGACCACAG	Map-based cloning	
MQJ2.R	TCAACAGACTCCGCATACT	Map-based cloning	
K9I9-1.F	TGGACTTGAATAGTTAGGCTGTCT	Map-based cloning	
K9I9-1.R	ATTACCAGTACTTAATAAAATGAT	Map-based cloning	
K4.542701.F	TGTTGCTGTGAGACTCTATCC	Mapping	
K4.542701.R	TAGACAAGCAGACTTCATGCC	Mapping	
AT4G01290.-1327	CGAGATTTCCAAGGTGTGAGTCC	Sequencing	*AT4G01290*
AT4G01290.-1106	GTTGGTTGGTTTATTACACTCTAGG	Sequencing	*AT4G01290*
AT4G01290.-679	TCAATTTTACCTTCCCCTTTGAGAG	Sequencing	*AT4G01290*
AT4G01290.-355	TTCATCTTTTCCCGATTTGAGG	Sequencing	*AT4G01290*
AT4G01290.+138	GCAGTTGCAATTGTTTTCAGGAAACC	Sequencing	*AT4G01290*
AT4G01290.+647	GTGGACTAGTATTCTGAATAGTTACC	Sequencing	*AT4G01290*
AT4G01290.+1174	TCTTGAATACTGCTCCATCACG	Sequencing	*AT4G01290*
AT4G01290.+1671	ATCACGCTCCAACAATTCCTGG	Sequencing	*AT4G01290*
AT4G01290.+2114	GAGTAAGAGAATTTGGGAATAGAGG	Sequencing	*AT4G01290*
AT4G01290.+2657	AGCTTTCCTCGATCTCGACTCC	Sequencing	*AT4G01290*
AT4G01290.+3132	ACGACTTGTTTGGGAAATGATAGGG	Sequencing	*AT4G01290*
AT4G01290.+3653	TCGGTGACAGCTATCATCCACC	Sequencing	*AT4G01290*
AT4G01290.+4154	ACCCATCAAAATACATGTCTTTTCC	Sequencing	*AT4G01290*
AT4G01290.+4643	ATGTAAACAACCAGATGCCGGG	Sequencing	*AT4G01290*
AT4G01290.ATG.attB1	GGGGACAAGTTTGTACAAAAAAGCAGGCTGTATGATGAGTATAGCAAATGAAC	Cloning Gateway	*AT4G01290*
AT4G01290.stop.attB2	GGGGACCACTTTGTACAAGAAAGCTGGGTGTTACCTGTAGCCAAACCCAAGG	Cloning Gateway	*AT4G01290*
AT4G01290.nostop.attB2	GGGGACCACTTTGTACAAGAAAGCTGGGTGTGACCTGTAGCCAAACCCAAGG	Cloning Gateway	*AT4G01290*
cbe1-1mut.F	ATGTACATTTGTAGGCGCCAC	Genotyping WiscDsLoxHs188_10F	*AT4G01290*
cbe1-1mut.R	CTTAATTCCCAACGGTTTTCC	Genotyping WiscDsLoxHs188_10F	*AT4G01290*
L4.WiscDsLoxHs.LB	TGATCCATGTAGATTTCCCGGACATGAAG	Genotyping	
SALK_038452_F	GAAATACGAAGCCCTCAGACC	Genotyping SALK_038452	*AT4G01290*
SALK_038452_R	GTATTGTTGGGGATGTTGGTG	Genotyping SALK_038452	*AT4G01290*
SALK_LBb1.3	ATTTTGCCGATTTCGGAAC	Genotyping	
GK-150H09_F	AGTATTCCATCCGTTCGATTCAC	Genotyping GK-150H09	*AT4G01290*
GK-150H09_R	AGAAACGAGAGTCCATAGAGAC	Genotyping GK-150H09	*AT4G01290*
GK_LB_o8474	ATAATAACGCTGCGGACATCTACATTTT	Genotyping	
BAK1-5.dCAPS.NruI.F	AGAGGGCTTGCGTATTTACATGATCATC	Genotyping *bak1-5*	*AT4G33430*
BAK1-5.dCAPS.NruI.R	GACCAATTGTCCCACGCACTG	Genotyping *bak1-5*	*AT4G33430*
Ubox.qF	TGCGCTGCCAGATAATACACTATT	RT-qPCR	*AT5G15400*
Ubox.qR	TGCTGCCCAACATCAGGTT	RT-qPCR	*AT5G15400*
At4g01290_qPCR_F_upstream	AGCACTGTTGCTTGACTTCG	RT-qPCR	*AT4G01290*
At4g01290_qPCR_R_upstream	GGCGATGAACTATAGTCATTCCG	RT-qPCR	*AT4G01290*
At4g01290_qPCR_F_downstream	TTGGGGATTCAGCAGAGGATGG	RT-qPCR	*AT4G01290*
At4g01290_qPCR_R_downstream	ACCCAAGGTCGAGTTCATGACC	RT-qPCR	*AT4G01290*
ACTIN2.qF	AGGTATCGCTGACCGTATGAGC	RT-qPCR	*AT3G18780*
ACTIN2.qR	ATCCACATCTGCTGGAATGTGC	RT-qPCR	*AT3G18780*
RBOHD.qF	ATGATCAAGGTGGCTGTTTACCC	RT-qPCR	*AT5G47910*
RBOHD.qR	GCAGTTCACCAACATGAACTGTCC	RT-qPCR	*AT5G47910*
cum1-1.F	AAGCCTAATTCAATAGAGAATCCGA	Genotyping	*AT4G18040*
cum1-1.R	GTCGGAAATAAAATAAAATCAAAAACCTAAGCT	Genotyping	*AT4G18040*
eIFiso4E-1.F	TTGACCCAATAGAGTCCAGAAAT	Genotyping	*AT5G35620*
eIFiso4E-1.R	CTCTCCAATCAAAGCCATCAACTA	Genotyping	*AT5G35620*
eIFiso4E-1.insert	GGTGCAGCAAAACCCACACTTTTACT	Genotyping	*AT5G35620*
eIF4Gmut.F	AGGTTCATGTTGATCAATGCC	Genotyping	*AT3G60240*
eIF4Gmut.R	GAACGCACCAGAGTGCTTATC	Genotyping	*AT3G60240*
eIF(iso)4G-1.F	TGATTGGTGAGCTTTTGAAGC	Genotyping	*AT5G57870*
eIF(iso)4G-1.R	CCAAGCTCCTCTACACACTGC	Genotyping	*AT5G57870*
eIF(iso)4G-2.F	AATGCAACAACAAGGTGAACC	Genotyping	*AT2G24050*
eIF(iso)4G-2.R	AAGAAGCTCGTACTTCTCCGG	Genotyping	*AT2G24050*
pat1-1.F	GGTTCCTTTCTCTTCAATCCG	Genotyping	*AT1G79090*
pat1-1.R	CGGAAGCTCTGTCGAGTATTG	Genotyping	*AT1G79090*
summ2-8.F	TACGCCATTCTTGTACCATCC	Genotyping	*AT1G12280*
summ2-8.R	CCACTAATGACGCTGAGCTTC	Genotyping	*AT1G12280*

Table S2. *CBE1* transgenics investigated.

Genetic background	Construct	Backbone
Col-0	*p35S-eGFP-cCBE1*	pK7WGF2.0
Col-0	*p35S-cCBE1-eGFP*	pK7FWG2.0
Col-0	*pCBE1-gCBE1-eGFP*	pGWB604
Col-0	*pUBI10-cCBE1-eGFP*	pUBC-GFP-Dest
bak1-5 mob7	*p35S-eGFP-cCBE1*	pK7WGF2.0
bak1-5 mob7	*p35S-cCBE1-eGFP*	pK7FWG2.0
bak1-5 mob7	*pCBE1-gCBE1-eGFP*	pGWB604
bak1-5 mob7	*pUBI10-cCBE1-eGFP*	pUBC-GFP-Dest
cbe1-1	*p35S-eGFP-cCBE1*	pK7WGF2.0
cbe1-1	*p35S-cCBE1-eGFP*	pK7FWG2.0
cbe1-1	*pCBE1-gCBE1-eGFP*	pGWB604
cbe1-1	*pUBI10-cCBE1-eGFP*	pUBC-GFP-Dest
